# Effect of Reservoir Heterogeneity on Polymer–Surfactant Binary Chemical Flooding Efficiency in Conglomerate Reservoirs

**DOI:** 10.3390/polym16233405

**Published:** 2024-12-03

**Authors:** Jianrong Lv, Guangzhi Liao, Weidong Liu, Xiaoguang Wang, Yuqian Jing, Hongxian Liu, Ruihai Jiang

**Affiliations:** 1 School of Engineering Science, University of Chinese Academy of Sciences, Beijing 100049, China; lvjianrong22@mails.ucas.ac.cn; 2 Institute of Porous Flow and Fluid Mechanics, Chinese Academy of Sciences, Langfang 065007, China; liaoguangzhi@petrochina.com.cn (G.L.); lwd69@petrochina.com.cn (W.L.); 3 Research Institute of Petroleum Exploration and Development, PetroChina, Beijing 100083, China; 4 Xinjiang Oilfield Company, PetroChina, Karamay 834000, China; wxguang@petrochina.com.cn; 5 PetroChina Exploration & Production Company, Beijing 100007, China; 6 College of Earth and Planetary Sciences, University of Chinese Academy of Sciences, Beijing 100049, China; jiangruihai23@mails.ucas.ac.cn; 7 Faculty of Petroleum, China University of Petroleum-Beijing at Karamay, Karamay 834000, China; lhx1979@petrochina.com.cn

**Keywords:** conglomerate reservoir, reservoir heterogeneity, polymer–surfactant binary flooding, oil flooding efficiency, nuclear magnetic resonance (NMR), CT scan

## Abstract

Reservoir heterogeneity significantly affects reservoir flooding efficiency and the formation and distribution of residual oil. As an effective method for enhancing recovery, polymer–surfactant (SP) flooding has a complex mechanism of action in inhomogeneous reservoirs. In this study, the effect of reservoir heterogeneity on the SP drive was investigated by designing core parallel flooding experiments combined with NMR and CT scanning techniques, taking conglomerate reservoirs in a Xinjiang oilfield as the research object. The experimental results show that inter-layer heterogeneity significantly affects water flooding efficiency and SP driving in low-permeability cores—the larger the permeability difference is, the more obvious the effect is—while it has almost no effect on high-permeability cores. The limited recovery enhancement in low-permeability cores is mainly due to the small percentage of contributing pores. When the permeability difference undergoes an extreme increase, the polymer molecular weight is biased towards higher values; when the polymer molecular weight is fixed, the recovery enhancement of low-permeability cores may be comparable to that of high-permeability cores when the permeability difference is extremely small. However, the recovery enhancement of the former is smaller than that of the latter when the permeability difference is extremely large. Due to intra-layer heterogeneity, there is a serious fingering phenomenon in the flooding stage, while in the SP flooding stage, recovery enhancement is most significant in the 5–20 μm pore range. This study provides an important geological basis for the rational development of a chemical flooding programme.

## 1. Introduction

Studying heterogeneity represents a fundamental aspect of reservoir characterisation. Investigating it is of paramount importance in elucidating the distribution of hydrocarbon water and residual oil, optimising development plans, and enhancing recovery rates [1,2,3]. The term ‘reservoir heterogeneity’ is used to describe the spatial inhomogeneity of physical parameters, such as porosity, permeability, saturation, and so forth. In the context of development and production, clastic reservoir heterogeneity is often classified into four categories, inter-layer, planar, intralevel, and microhomogeneity, in order of increasing severity [4]. In this regard, a great deal of work has been carried out by other scholars using a variety of research methods and techniques. These include the integrated geological analysis method using well logging seismic data [5,6], the heterogeneous synthetic index [7], stratigraphic stratigraphy [8], and others. Additionally, the experimental method has been employed using techniques such as nuclear magnetic resonance [9], electron microscope scanning [10], CT scanning [9], numerical simulation [11], and reservoir geological modelling [12]. These methods demonstrate the laws of oil and gas transport and storage in practical applications, thereby providing a theoretical basis and solutions for developers in addressing the complex issues of reservoir heterogeneity. The results achieved thus far are promising.

The formation of reservoir heterogeneity is the result of a complex interplay between the depositional environment, diagenesis, and late tectonic action. The previous studies have elucidated the underlying causes of various challenges encountered during oilfield development, including local residual oil enrichment [13], inter-layer interference [14], and premature water flooding [15]. These issues are attributed to reservoir heterogeneity. The formation of conglomerate reservoirs has resulted in the development of a clastic system characterised by a complex pore structure and strong heterogeneity. This is attributed to its proximal origin, the presence of multiple water systems, and the variable paleo-foothill geography, along with its complex transport mechanisms and unstable hydrodynamic conditions [16]. In order to guarantee the uninterrupted, effective, and stable field production, polymer and SP binary composite flooding were introduced. Deng et al. employed CT scanning to observe the processes of water and polymer flooding in conglomerate reservoirs in real time, with the aim of analysing the mechanism of conglomerate flooding [17,18,19,20]. Liu et al. chose natural cores with three typical pore structures to study the effect of structural differences on the recovery enhancement of binary composite flooding at three scales: the pore, the core, and the field [21]. Wei et al. added velvet capsule fluid into the polymer. A large number of vesicles in an inhomogeneous reservoir can block the hypertonic channel, prompting the repellent medium to enter the low-permeability channel, and further improving the polymer flooding recovery rate [22]. Tan Long et al. proposed a graded chemical injection scheme, which allowed for the chemical to enter the pore space sequentially at different scales, and achieved the graded mobilisation of residual oil after water flooding in conglomerate reservoirs [23]. Nevertheless, most current research on enhancing recovery in conglomerate reservoirs concentrates on optimising system formulations and modifying the injection scheme, with minimal attention devoted to investigating the impact of reservoir heterogeneity on chemical flooding.

Conglomerate reservoirs’ complex depositional conditions are the primary determinants of significant reservoir heterogeneity. The pore–throat distribution of these reservoirs exhibits a considerable range characterised by pronounced microporous structure heterogeneity. According to our analysis and the evaluation of reservoirs where chemical flooding has been implemented, we found that under the condition of the same permeability, the microscopic pore–throat structure of the same or different lithologies in the same block varies significantly, and optimising the chemical flooding formulation system according to permeability causes problems. Therefore, for conglomerate reservoirs, studying the effect of increased heterogeneity on chemical drive flooding efficiency is the key prerequisite for efficient reservoir development.

## 2. Geological Overview of Oil Reservoirs

The oil reservoir of the Badaowan Formation in the Xinjiang Oilfield in Seventh Block is located in the central part of the Ke-Wu Fault Line on the northwestern margin of Junggar Basin, which is a monoclinic dip from north to southeast and southwest. It becomes progressively steeper along the dip direction and is an overall tectonic rocky oil reservoir. From the bottom to the top, its lithology exhibits a gradational transition from conglomerate to fine sandstone. The majority of the minor layers contain bottom conglomerate, and the grain size is characterised by positive rotation. Its reservoir lithology is characterised by the prevalence of medium and coarse sandstone and conglomerate; the oil content of the former is the most optimal, followed by that of the latter. Based on the field data and the existing studies [24], the reservoir’s average porosity is 20.0%, and its average permeability is 124.6 mD, making it a medium-porosity, medium-permeability reservoir. The pore–throat structure is intricate, comprising primarily intergranular solution holes and primary intergranular holes, followed by intragranular solution holes and microcracks. It is predominantly characterised by bent and lamellar throats.

**Figure 1:**
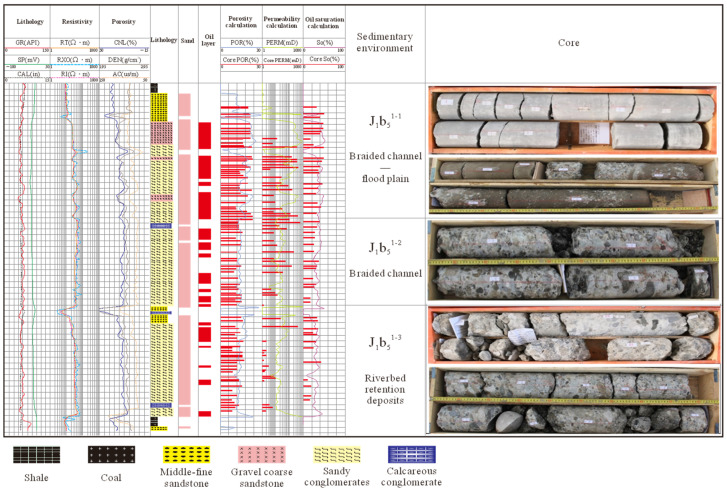
A column diagram of the sedimentary lithology of the J_1_*b*_5_ layer in the Badaowan Formation in the Seventh Block.

The Badaowan Formation consists of J_1_*b*_5_, J_1_*b*_4_, J_1_*b*_3_, J_1_*b*_2_, and J_1_*b*_1_ from the bottom up, of which J_1_*b*_5_ can be further divided into three minor layers—J_1_*b*_5_^1-3^, J_1_*b*_5_^1-2^, and J_1_*b*_5_^1-1^—which are the main oil-bearing formations. The heterogeneity coefficient of permeability is defined as the ratio of the maximum value of permeability (*K*_max_) to its average value (*K*) in a selected well section or single sand layer. This can be expressed as *T*_k_ = *K*_max_/*K*. The difference in level reflects the absolute difference in permeability across different locations; a large difference indicates that permeability is unevenly distributed. The coefficient of variation indicates the relative degree of fluctuation in permeability, with a high value implying greater differences in permeability and a more heterogeneous reservoir. These two indicators assist in assessing the spatial distribution of permeability, which is of great significance in oil and gas reservoir development and fluid flow analysis. The analysis of the reservoir heterogeneity coefficients (Table 1) and sedimentary lithology histograms (Figure 1) shows that J_1_*b*_5_^1-1^ has complex sedimentary phases and lithological variations, with large gradation and syncline coefficients and the development of hypertonic channels, as well as poor reservoirs. The J_1_*b*_5_^1-2^ and J_1_*b*_5_^1-3^ formations are dominated by fluvial stagnant gravels and coarse-grained deposits. Despite the relatively poor physical properties of these formations, the absence of hyper channels and the low gradient and breakthrough coefficients indicate that they are not susceptible to this particular type of geological phenomenon. The J_1_*b*_5_^1-3^ formation is dominated by stagnant riverbed deposits, with a complex pore structure, large changes in particle size, large coefficients of variation, and the strongest inhomogeneity. A number of chemical flooding development experiments, including polymer and SP binary composite flooding, have been conducted in the target reservoir within this study area. These have yielded positive outcomes, substantiating the considerable potential of chemical flooding in conglomerate reservoirs for enhancing recovery rates. Nevertheless, with the progressive advancement of chemical flooding techniques in conglomerate reservoirs, a number of geological challenges have emerged. Among these issues, the impact of reservoir heterogeneity on flooding efficiency is particularly noteworthy. It is therefore essential to undertake an evaluation of reservoir heterogeneity in order to enhance chemical flooding efficiency.

## 3. Experimental Methods and Procedures

### 3.1. Experimental Principle

The CT technique, or ray-based computed tomography, enables the acquisition of images of various fluids present within the core at different stages of the flooding process. The beam generated by the X-ray tube in the CT machine is irradiated from multiple directions along the selected object fault level. The quantity of X-rays transmitted is gauged and digitised, and the absorption coefficients per unit volume of tissue at that level are calculated. These can then be formed into different numerical matrices. Through the machine’s high-speed computer, which carries out digital–analogue conversion, the reconstructed image can be displayed on the screen, or captured as a photograph. Additionally, the image can provide the X-ray attenuation coefficient for each pixel [25].

In the context of core flooding, using nuclear magnetic resonance (NMR) enables the acquisition of data pertaining to porosity and pore size distribution. The protons in the fluid are polarised in the magnetic field of the instrument. Upon magnetic field withdrawal, the protons begin to return to their original equilibrium position in a process known as relaxation; the time taken for this process to occur is referred to as relaxation time (*T*_2_). Once the porous medium is completely saturated with water, the *T*_2_ value of a single pore is found to be proportional to the ratio of surface area to the volume of the pore. This ratio serves as a measure of the pore size, with the observed *T*_2_ distribution of all pores representing the pore size distribution of the rock [26]. There are three fluid relaxation behaviours at the pore throat: free relaxation *T*_2_B, surface relaxation *T*_2_S, and diffusion relaxation *T*_2_D. In the case of crude oil-saturated cores, the *T*_2_ relaxation time is primarily contingent upon the surface relaxation time, *T*_2_S, which can be expressed as follows:

(1) $${\left(\frac{1}{T_{2}}\right)}_{S}=\rho_{2}\;{\left(\frac{S}{V}\right)}_{\;pore}$$

(2) $$\frac{S}{V}=\frac{F_{S}}{r}$$

where *ρ*_2_ is the transverse surface relaxation rate, µm/ms; *S/V* is the ratio of pore surface area to volume; *F*_S_ is the geometry factor; and *r* is the pore radius, µm. Equations (1) and (2) can be linked to yield the following result:

(3) $${\left(T_{2}\right)}_{S}=\frac{1}{\rho_{2}F_{S}}r$$

Assuming 1/(*ρ*_2_*F*_S_) = *C*, Equation (3) can be transformed into *T*_2_ = C × r. Consequently, following the derived coefficient value C, the NMR *T*_2_ distribution can be transformed into a pore radius distribution [27].

### 3.2. Selection of Experimental Samples

**Figure 2:**
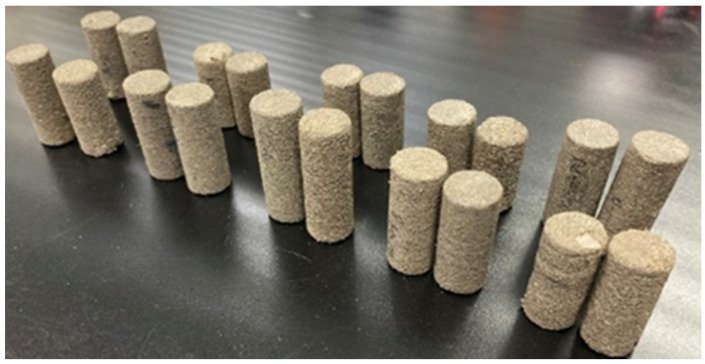
Photographs of cores from parallel experiments.

Given the considerable heterogeneity of conglomerate reservoirs, the samples employed in experiments were natural conglomerate cores extracted from the Badaowan Formation reservoir in the Seventh Block. This approach was taken in order to restore the permeability characteristics of the conglomerate reservoirs as closely as possible to their natural state (Figure 2).

The core used for computed tomography (CT) scanning is a natural outcrop core with a single-layer core size of 45 mm × 45 mm × 60 mm. Its longitudinal composition comprises three interconnected rectangular cores of equal thickness separated by specialised T material. The application of circumferential pressure enables the injection fluid to be effectively prevented from flowing along the interstitial spaces between individual layers, while ensuring that the longitudinal permeability of the core remains uncompromised. The parameters of the multilayer model are presented in Table 2.

**Figure 3:**
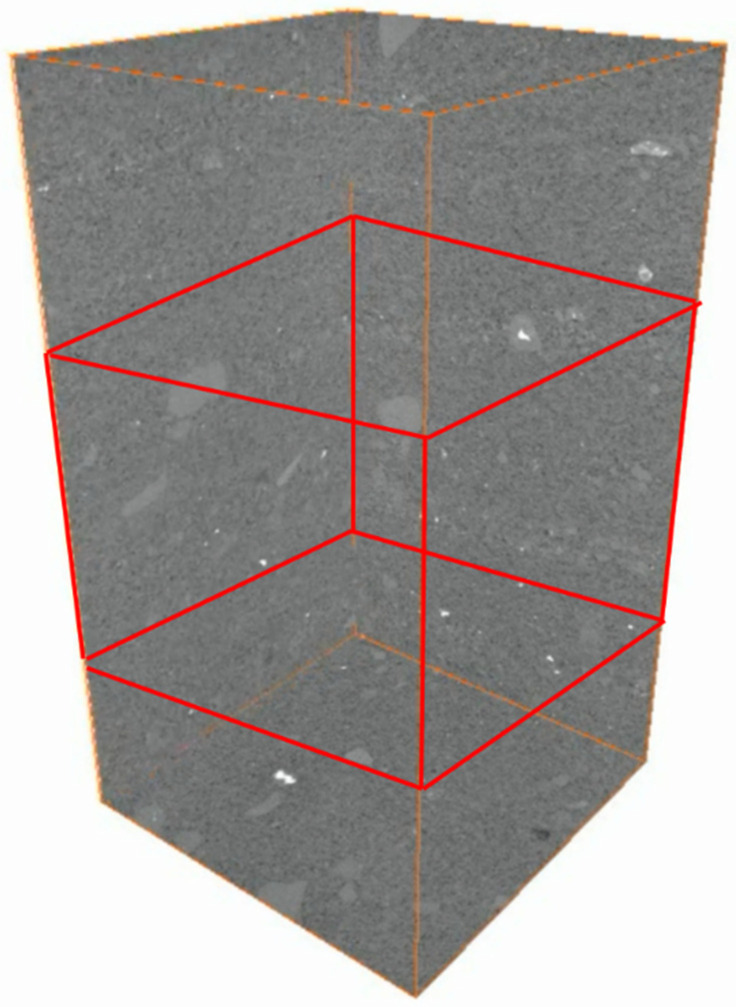
CT scan of rectangular core (resolution 22.5 µm).

**Figure 4:**
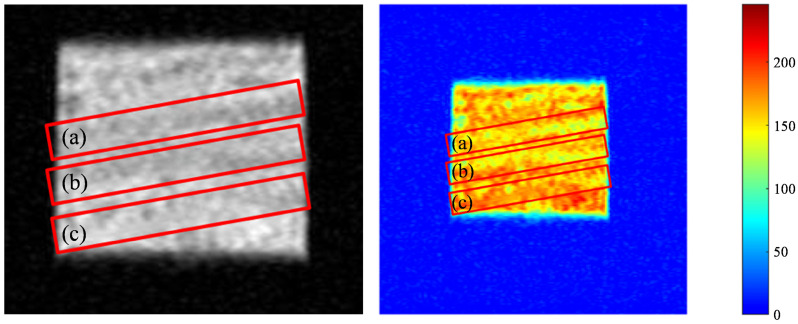
MRI scans of orthoclase cores: (a) first; (b) second; and (c) third layers.

Intra-layer heterogeneity NMR experiments were conducted on full-diameter cores, which were cut into rectangular cores measuring 50 mm × 50 mm × 110 mm. Additionally, CT scans were performed and are presented in Figure 3. As indicated by the CT scanning results, the section displaying clear rhythmicity (enclosed within the red cube in Figure 2) was subsequently divided into 45 mm × 45 mm × 45 mm square cores and subjected to saturated water magnetic resonance imaging (MRI). The resulting data are presented in Figure 4. MRI was conducted using a spin-echo sequence and fast spin-echo series to create a map. In the course of the flooding experiment, the distinction between oil and water signals was made more effectively by using heavy (D_2_O) instead of ordinary water (H_2_O). This was because the former has no NMR signals, which meant that all the signals collected were oil signals. The distribution and transport characteristics of the crude oil in the rock core were then analysed. The brightness of the map correlates with the number of hydrogen-containing signals, and three layers, each with a thickness of 10 mm, were selected for analysing the impact of intra-layer heterogeneity on polymer repulsion through NMR scanning.

### 3.3. Experimental Materials and Instruments

The experimental oils were analogue oils formulated using 5# and 26# white oils, with a viscosity of 26.6 mPa-s. These were augmented with CT enhancers for scanning. Experimental water was simulated stratum water, the relevant properties and composition of which are presented in Table 3.

The screening of the binary composite flooding chemical system was based on the results of previous experiments that were deemed relevant to the current study. Polyacrylic acid amine (HPAM) from Beijing Hengju Chemical Co., Ltd. (Beijing, China) was selected as the polymer of choice, with relative molecular masses of 500 × 10^4^, 800 × 10^4^, 1200 × 10^4^, and 1500 × 10^4^, respectively. The surfactant selected for use was petroleum sulfonate KPS provided by Karamay Jinta Company (Karamay, China), with a mass active substance fraction of 20%. The polymer molecular weights employed in the core parallel flooding experiments were distinct, with the objective of conducting disparate molecular weight flooding experiments under a uniform gradient. This approach was undertaken to analyse the ripple pattern of SP flooding under heterogeneous conditions and to optimise chemical formation under heterogeneous conditions, with the aim of enhancing flooding efficiency. The specific parameters are outlined in Table 4.

**Figure 5:**
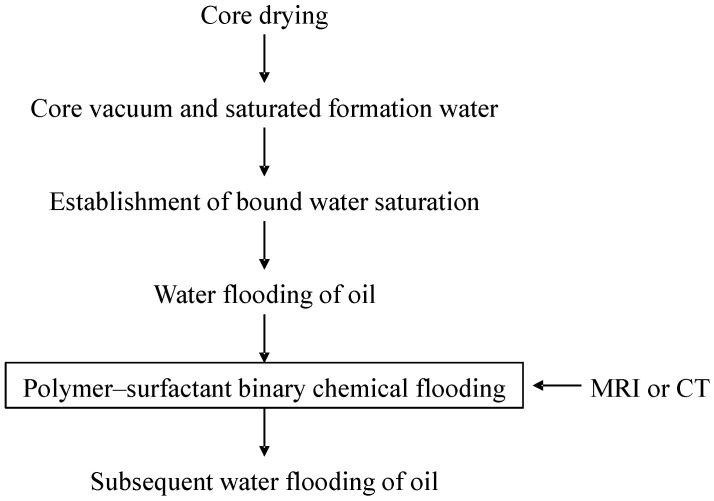
Flowchart of experimental steps.

The central component of the parallel driving experiment comprises two parallel core clamps. The driving apparatus utilises the SP-5000 oil–water flooding system produced by QUIZIX Company of the United States (Broken Arrow, OK, USA). The overlying pressure apparatus employs a TC-1000 automatic perimeter pressure pump. Nuclear magnetic resonance (NMR) experimental instrumentation comprised a high-temperature and pressure NMR online flooding nuclear magnetic imaging system (SPEC035, Beijing Spectrum Science and Technology Development Co., Ltd., Beijing, China). The experiments were conducted with two parallel core clamps, the flooding device for which was the ISCO100DX oil–water flooding system produced by U.S. Teledyne Company (Thousand Oaks, CA, USA). The overlying pressure device was an automatic perimeter pressure pump. The computed tomography (CT) scanning system employed the LIGHTSPEED 8-slice spiral CT from General Electric (GE), Boston, MA, USA, which utilises single-slice scanning. The tube voltage is 120 kV, the tube current is 130 mA, the scanning layer thickness is 1.25 mm, the scanning layer spacing is 5.000 mm, there are four rows of detector plates, the scanning time is 0.5 s/360°, and the flooding system employs a Quizix SP-5000 high-pressure metering pump, Chandler, AZ, USA. The experimental steps are shown in Figure 5.

### 3.4. Displacement Experiment

**Figure 6:**
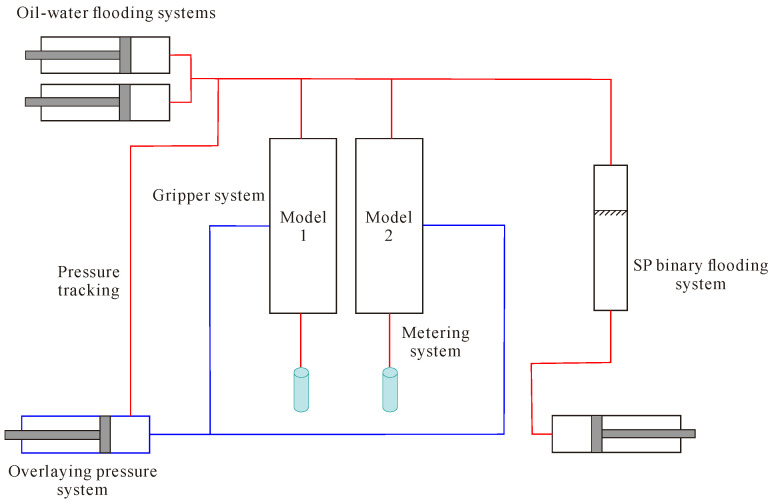
Displacement experiment process.

The experimental steps are as follows (Figure 6):

(1) Core vacuum and saturated formation water.
  The cores were subjected to a drying process, followed by a gassing procedure to determine their porosity and permeability. Subsequently, they underwent vacuum treatment and saturation with a configuration of simulated formation water.
(2) Establishment of bound water saturation.
  The core evacuated the saturated simulated formation water, driven with oil at a speed of 0.02 mL/min until the outlet section no longer produced water. The displacement speed was increased to 0.2 mL/min; to 0.3 mL/min; to 0.4 mL; and to 0.5 mL/min to drive the water until the outlet section no longer produced water. In this way, we can calculate irreducible water saturation.
(3) Water flooding of oil.
  Water flooding was simulated using constant velocity drive—with a flow rate of 0.2 mL/min for two cores in parallel and 0.4 mL/min for four cores in parallel—until the water content of three experimental points of high-permeability cores reached 98% and above consecutively. Thereafter, water flooding was terminated.
(4) Polymer–surfactant binary chemical flooding.
  A configured binary system was employed to displace 0.7 PV, which, in turn, resulted in flooding cessation.
(5) Subsequent water flooding of oil.
  Simulated formation water flooding was employed until the water content reached 98 per cent or above at three consecutive experimental points in the high-permeability core, at which point subsequent water flooding was terminated.

### 3.5. Nuclear Magnetic Experiment

**Figure 7:**
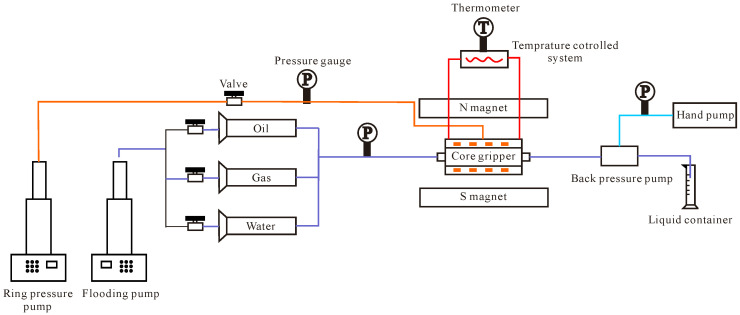
Nuclear magnetic resonance experiment process.

The experimental steps are as follows (Figure 7):

(1) Core vacuum and saturated formation water.
  The cores were subjected to a drying process, followed by a gassing procedure to determine their porosity and permeability. They were then evacuated of the saturated configurations of simulated formation water, and finally tested for *T*_2_ distribution using nuclear magnetic resonance (NMR).
(2) Establishment of bound water saturation.
  For drying, the core was emptied of saturated heavy water (D_2_O) to configure simulated formation water, and oil was driven at a speed of 0.02 mL/min until the outlet section did not produce water. The displacement speed was then increased to 0.2 mL/min; to 0.3 mL/min; to 0.4 mL/min; and to 0.5 mL/min to drive the water until the outlet section no longer produced water. Irreducible water saturation was calculated, and the core *T*_2_ distribution was tested by nuclear magnetic resonance.
(3) Water flooding of oil.
  We employed a 0.2 mL/min water flooding rate in heavy water (D_2_O) configuration to simulate formation water flooding until the water content of three experimental points in high-permeability cores reached 98% and above consecutively, at which point water flooding was terminated and the NMR testing of core *T*_2_ distribution commenced.
(4) Polymer–surfactant binary chemical flooding.
  An SP binary system configured with heavy water (D_2_O) was employed to flood crude oil for 0.7 PV, which, in turn, terminated the flooding process.
(5) Subsequent water flooding of oil.
  A heavy water (D_2_O) configuration was employed to simulate formation water flooding until the water content reached 98% and above at three consecutive experimental points in the high-permeability core. At this point, subsequent water flooding was terminated, and the T_2_ distribution of the core was tested by nuclear magnetic resonance (NMR).

### 3.6. CT Scan

**Figure 8:**
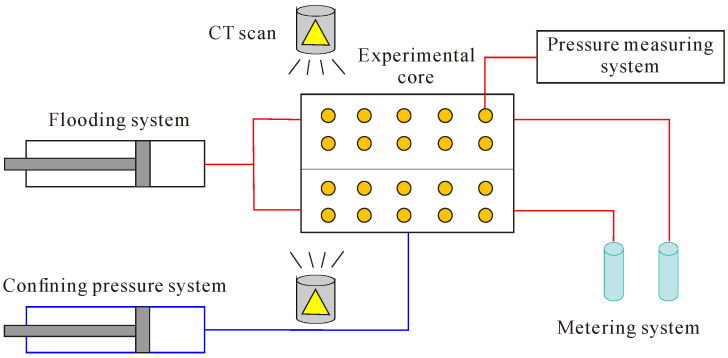
Experimental procedure of CT scanning.

The experimental steps are as follows (Figure 8):

(1) The cores were dried and placed in a holder for scanning.
(2) The cores were evacuated and saturated with formation water for saturated water core scanning.
(3) Oil flooding was conducted to establish bound water saturation.
(4) In the water flooding oil experiment, the flooding method was used at a constant speed of 0.3 mL/min until the water content reached 98%.
(5) In the polymer–surfactant binary chemical flooding experiment, an SP drive of 0.7 PV was used to achieve a water content of more than 98%. CT scanning was carried out to monitor oil–water saturation change during the flooding process. This was then combined with information on the water content, oil drive efficiency, and recovery rate to obtain the characteristics of water and polymer flooding in the core.

## 4. Results and Discussion

### 4.1. Influence of Inter-Layer Heterogeneity on Flooding Effectiveness

#### 4.1.1. Core Parallel Flooding Experiments

**Figure 9:**
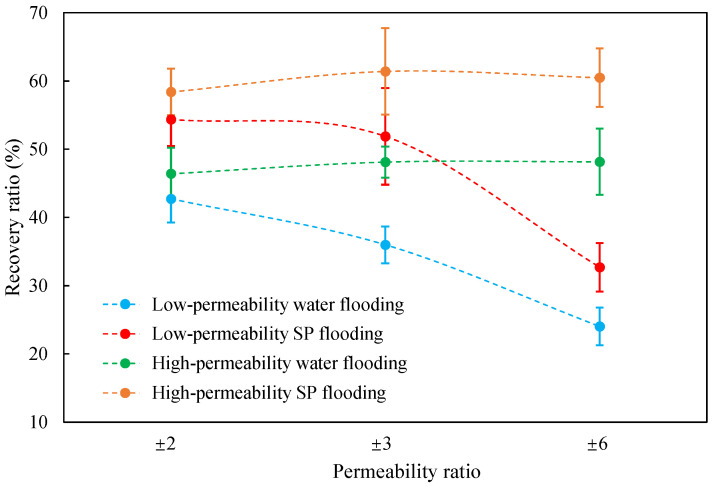
Variation curve of recovery rate with permeability polarity for combined model.

The term ‘inter-layer heterogeneity’ is used to describe the variability observed between multiple reservoirs in the longitudinal direction. It represents a significant basis for evaluating reservoirs, discovering production potential, and predicting ultimate recovery [28]. As presented in Table 5, the results of the parallel core flooding experiments demonstrate that as the permeability difference undergoes an extreme increase, the combined model exhibits a decline in both water flooding efficiency and SP post-flooding recovery. For the combined model, the change curve of the recovery rate (Figure 9) demonstrates that the recovery rate of the high-permeability cores is largely unaffected by an extreme permeability difference. Conversely, the water and SP flooding efficiencies decrease significantly with an extreme increase in permeability difference in the low-permeability cores. Therefore, it can be concluded that the decrease in the final recovery rate of the combined model is primarily due to the low-permeability cores. In the case of the low-permeability cores, the production time at the low-water-content stage is relatively brief, with that of the core reaching its peak with rapidity. As a consequence, the recovery rate is unable to be augmented due to the extended high water content stage. In contrast, for the high-permeability cores, the production time is considerably longer, with the recovery rate increasing in a gradual and sustained manner (Table 6). This suggests that in the combined model, the hypertonic core assumes a dominant role, while the low-permeability core exhibits a limited wave volume and low flooding efficiency.

Table 7 illustrates the extent of recovery enhancement achieved through SP flooding, as observed in each experimental group. In the case of extreme permeability difference determination, the larger the permeability difference is, the more inclined the polymer molecular weight with the largest recovery enhancement in the low-permeability cores is to a higher value. This is manifested in the fact that the 800 × 10^4^ molecular weight + 0.2% KPS binary system showed the greatest recovery enhancement at an extreme permeability difference of two, and the 1200 × 10^4^ molecular weight + 0.2% KPS and 1500 × 10^4^ molecular weight + 0.2% KPS and 0.2% KPS binary systems showed the greatest recovery enhancement at extreme permeability differences of three and six, respectively. The 0.2% KPS binary system showed the greatest recovery enhancement. When the polymer molecular weight of the binary system was determined, the recovery improvement in the low-permeability core is greater than, or about the same as that of the high-permeability core when the permeability difference was extremely small (two or three) and less than that of the high-permeability core when the permeability difference was extremely large (six); the recovery improvement in the binary system of polymer flooding with a molecular weight of 1500 × 10^4^ + 0.2% KPS is less than that of the low- and high-permeability cores. The parallel core flooding experiments clarified that intra-layer heterogeneity mainly affects the recovery of the low-permeability cores, and the larger the permeability difference is, the more pronounced the effect is. For the case of very poor permeability, flooding is possible by the appropriate selection of a high-molecular-weight binary system, which is able to improve the recovery rate of low-permeability cores by a small margin. However, the flooding effect of the 1500 × 10^4^ molecular weight + 0.2% KPS binary system is not obvious. It is therefore necessary to begin with low-permeability cores in order to ascertain the reasons for their reduced recovery in non-homogeneous conditions.

#### 4.1.2. Nuclear Magnetic Resonance Analysis of Parallel Cores

**Figure 10:**
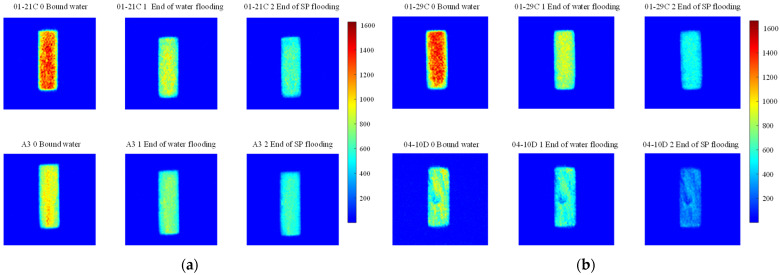
MRI dynamic monitoring map of core flooding (along flooding direction): (**a**) Combinations 11 and (**b**) 12.

**Figure 11:**
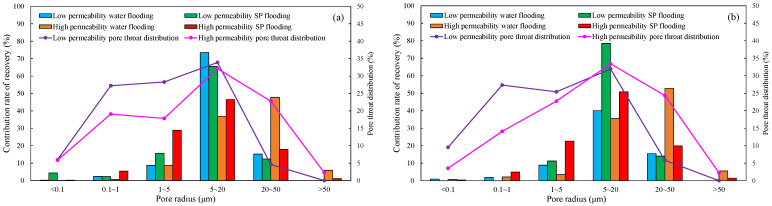
Contribution of different pore size distributions to recovery rate: (**a**) Combinations 11 and (**b**) 12.

NMR analyses were performed on assemblies 11 and 12, and the pore structure characterisation parameters were calculated from the *T*_2_ distribution curves (Table 8). The comparative analysis of the core flooding MRI dynamic monitoring map (Figure 10) indicates that the saturation of high-permeability cores, 01-21C and 01-29, decreased significantly following SP flooding, whereas the saturation of low-permeability core A3 and the decrease in saturation observed in the 04-10D core were less pronounced. There are fewer laminations in the low-permeability cores, and the number of laminations in the high-permeability cores decreases significantly with water and SP flooding. The analysis of pore space distribution and the recovery rate (Figure 11) at the water flooding stage revealed that the pore space contributing the most to the water flooding recovery rate is 5–50 μm. This pore space accounts for approximately 36% of the total in the low-permeability cores, while in the high-permeability cores, it reaches 55%. The primary distinction between the two lies in the distribution of pore space within each core. The distribution of pore spaces of 20–50 μm in the low-permeability cores accounts for only approximately 5%, whereas the proportion in the high-permeability cores is greater than 20%. Therefore, the greater water flooding efficiency observed in the high-permeability cores can be attributed to the differing proportions of pore space contributing to the recovery rate. It can therefore be concluded that the higher water flooding efficiency observed in the high-permeability core is a consequence of the differing proportions of pore space contributing to the recovery rate. To illustrate, the water flooding recovery rate contribution of the 5–50 μm pore space in the low-permeability core of Combination 11 is 88.63%, the pore throat percentage is 33.92%, and 20–50 μm accounts for a mere 4.64%. The equivalent rate in the high-permeability core reaches 84.71%, with a pore throat percentage of 54.95%. In contrast, a pore throat of 20–50 μm accounts for 22.80%, representing a significantly higher proportion than that observed in the low-permeability core.

In the SP flooding stage, the recovery of all the pores with a radius greater than 5 μm is significantly enhanced, with the greatest contribution observed in the 5–20 μm range. For the low-permeability cores with less than 5 μm, the proportion of pores with a radius of 5–20 μm is relatively low, and the SP flooding of the high-permeability cores with a pore radius of 1–5 μm is also considerably improved. Conversely, the contribution of the low-permeability cores to pore recovery is relatively small. To illustrate, Combination 12, comprising a low-permeability core with a pore space greater than 5 μm, exhibits an SP flooding recovery rate contribution of 92.57%, with the pore space accounting for 37.77%. Similarly, the high-permeability core with a pore space greater than 5 μm demonstrates an SP flooding recovery rate contribution of 72.12%, with the pore space accounting for 59.97%. The percentages of hypertonic and hypotonic cores for the pores of 1~5 μm are not significantly different. However, the contribution of the SP flooding recovery rate in the hypotonic cores is 11.27%; in the hypertonic cores, it is approximately twice as much, reaching 22.56%. Therefore, the principal factor contributing to the slight increase in recovery in the low-permeability cores is the relatively small proportion of pore space that significantly influences recovery.

### 4.2. Influence of Intra-Layer Heterogeneity on Flooding Effectiveness

#### 4.2.1. CT Scan Analysis

**Figure 12:**
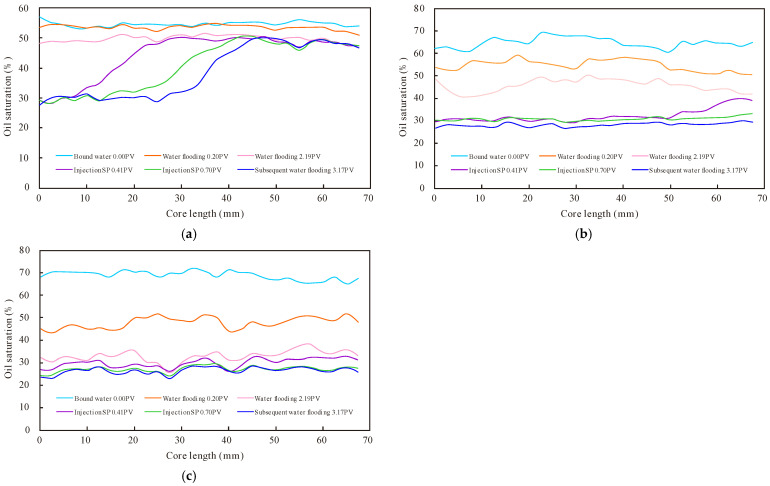
Change in oil saturation during flooding process: (**a**) low-; (**b**) medium-; and (**c**) high-permeability layers.

Intra-layer heterogeneity refers to the variation in vertical reservoir properties within a single sand formation. This phenomenon plays a pivotal role in controlling and influencing the injectant swept volume and residual oil distribution within a given sand formation. Intraformational non-homogeneous oil layers are frequently observed in conglomerate reservoirs. They are typically characterised by the presence of low-, medium-, and high-permeability orthorhombic rhythms. A physical simulation experiment of the SP binary composite drive for enhanced recovery was carried out on a non-homogeneous oil layer within the orthorhombic layer using CT scanning technology. By analysing the oil driving efficiency of each layer (Table 9) and the change in oil saturation in the process of substitution (Figure 12), it can be seen that there is a significant difference in the use of the water drive on different layers, with a high degree of use in the top seepage layer, only partial use in the middle seepage layer, and basically no use in the bottom seepage layer; the SP drive significantly improves the recovery and has a significant effect on the middle and bottom seepage layers.

**Figure 13:**
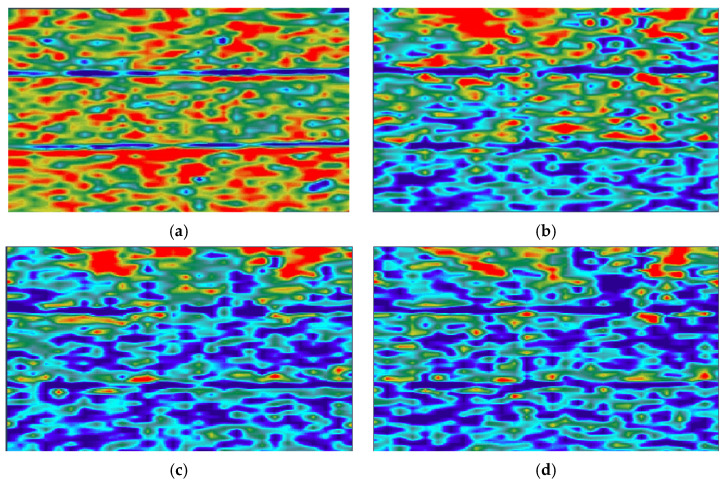
Reconstructed section of CT scan for heterogeneity modelling: (**a**) bound water; (**b**) after water flooding; (**c**) after SP flooding; and (**d**) end of flooding.

The distribution of oil saturation in the heterogeneous model within the layers (Figure 13) demonstrates that oil saturation within the core decreases during water flooding. However, the degree of rippling is markedly uneven, and there is a significant fingering phenomenon. The rate of decline in oil saturation in the high-permeability layer is rapid, while the rate of decline in oil saturation in the middle- and low-permeability layers is relatively slow. The positive rhythm model is not utilised effectively, and the remaining oil is concentrated in the entire low-permeability layer and the middle portion of the middle-permeability layer. This is due to the injection of water into the high-permeability layer with low resistance at the bottom, which results in the upper low-permeability layer almost not being washed. The extraction end is washed by the action of capillary pressure, which results in the middle-permeability layer being washed at the two ends. The remaining oil is mainly concentrated in the middle portion of the layer. In the stage of SP flooding, the residual oil that is difficult to replace after water flooding is effectively mobilised. The residual oil left in a small area still cannot be effectively mobilised. The elevated viscosity of the polymer in the binary system results in a pronounced surge in viscous resistance within the high-permeability layer. Additionally, a pressure gradient is observed between the high-permeability layer and the medium- and low-permeability layers. When the pressure gradient reaches a point where it exceeds the capillary pressure and viscous resistance of the polymer in vertical seepage, the binary system bypasses the high-permeability layer and enters the medium- and low-permeability layers, effectively enlarging the swept volume of injected fluid. Furthermore, the elevated viscosity of the polymer markedly enhances the oil–water flow rate ratio, effectively circumventing the fingering and tampering phenomena that arise during the flooding process. This allows for the more uniform distribution of the injected fluid towards the outlet and facilitates near-piston flooding, which mitigates the ineffective circulation of injected water and plays a beneficial role in anatomical conditioning.

#### 4.2.2. Nuclear Magnetic Resonance Analysis

**Figure 14:**
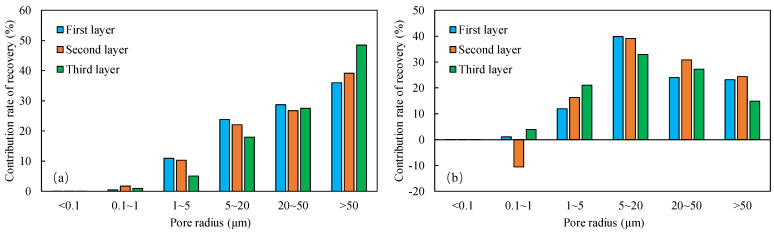
Contribution of enhanced recovery with different pore water flooding (**a**) and SP flooding (**b**) experiments.

The results of the NMR analysis of the effect of intra-layer heterogeneity on SP flooding are shown in Table 10. From these, it can be seen that the oil flooding efficiency of water in the first hypertonic layer is 55.8%, the recovery rate after SP flooding is 63.1%, and SP flooding is improved by 7.3%; the oil flooding efficiency of water in the second hypertonic layer is 65.6%, the recovery rate after SP flooding is 60.4%, and SP flooding is improved by 5.1%; and the oil flooding efficiency of water in the third hypertonic layer is 39.0%, the recovery rate after SP flooding is 52.1%, and SP flooding is improved by 13.1%. The oil recovery rate of the third layer is markedly inferior to those of the first and second layers. This is evidenced by the findings of the preceding section, which indicate that the permeability of the third layer is relatively low. From the data shown in Figure 14a, it can be observed that the recovery rate contribution increases with an increase in pore radius. The comparison of the change in *T*_2_ in each layer (Table 11), with the contribution of using different pore SP flooding experiments to improve the recovery rate (Figure 14b), indicates that for the three layers and despite the observed differences in permeability, the percentage contribution of each pore radius remains relatively consistent. The third layer of core SP flooding demonstrates a notable enhancement in the recovery rate, particularly for the pore radii exceeding 1 μm. The first and second layers exhibit a discernible improvement in recovery rate, with the pore radii ranging from 5 to 20 μm. The recovery rate of the third layer after SP flooding of the cores with a pore radius greater than 1 μm increases significantly. The recovery rate of the first and second layers with a pore radius greater than 5 μm sees a certain increase; the pores of the three layers with 5–20 μm pores exhibit the largest increase.

### 4.3. Comparative Study of the Influence of Heterogeneity

The analysis of the aforementioned experiments indicates that reservoir heterogeneity primarily influences the recovery of the low-permeability section. Conversely, inter- and intra-layer heterogeneity do not exert the same degree of influence on the low-permeability section. The presence of inter-layer heterogeneity gives rise to significant variations in the water flooding effect across layers with disparate permeability polarities. This phenomenon is particularly evident in the case of the high-permeability layer undergoing single-layer breakthrough, while the low-permeability layer exhibits limited mobility, resulting in the formation of residual oil. In the SP flooding stage, the action of polymers in the binary system resulted in an improvement in the recovery of the low-permeability cores. However, the extent of this recovery was constrained by the relatively limited contribution of the pore space to overall recovery. The percentages of pore space occupied by a given radius in the high- and low-permeability sections are similar within a single layer. Consequently, the mechanism of influence of intra-layer heterogeneity is analogous to that of inter-layer heterogeneity. The occurrence of the former can result in the formation of severe fingering during the flooding phase, which manifests as highly uneven ripples. The high viscosity of the polymer markedly enhances the oil–water flow ratio, which can be instrumental in regulating dissection. However, for the low-permeability portion, flooding is only efficacious in replacing contiguous residual oil after the water drive. Furthermore, the small amount of residual oil remains unexploited. Although the two types of heterogeneity affect the low-permeability core in different ways, from a microscopic point of view, the pore radius that contributes most to recovery enhancement is 5–20 μm. Therefore, the analysis of the flooding effect of different SP formulations can be initiated with a 5–20 μm pore space, with the objective of maximising the mobilisation of residual oil in this part.

In summary, for reservoirs with strong heterogeneity, the SP flooding programme can be adjusted according to the different types of heterogeneity and the influence of different permeability cores, which can further improve reservoir recovery.

## 5. Conclusions

(1) In the case of heterogeneity, it was observed that as the permeability ratio increases a lot, the molecular weight of the polymer that exhibits the greatest improvement in recovery in the low-permeability cores also increases. This is in accordance with the findings regarding the permeability ratio. Once the molecular weight of the polymer in the binary system has been determined, it becomes evident that in the cases where the permeability ratio is low, the improvement in core recovery is significantly greater than, or comparable to that of the high-permeability core. Conversely, in instances where the ratio is high, the improvement in core recovery is less pronounced than that of the high-permeability core. Additionally, the 1500 × 10^4^ molecular weight + 0.2% KPS binary system flooding recovery improvement is observed to be less pronounced in the cases of low permeability than in the instances of high permeability.
(2) Inter-layer heterogeneity has a significant impact on the efficacy of water and SP flooding in the low-permeability cores. The effect is most pronounced when the permeability ratio is large, whereas it is almost negligible in the high-permeability cores. The relatively low recovery rate observed in the low-permeability cores can be attributed to the limited pore space available for recovery, with the majority of contribution coming from the crude oil present within the pore spaces of 5–20 μm.
(3) Intra-layer heterogeneity resulted in significant fingering phenomena in the water drive, with the remaining oil concentrated in the low-permeability layer and the middle portion of the middle-permeability layer. In the SP drive stage, despite the residual oil being replaced with water, it was effectively utilised, with the majority having distributed itself on a continuous sheet. Pore oil with a diameter of 5–20 μm was the primary contributor to the SP drive, facilitating an improvement in the recovery rate.

## Figures and Tables

**Table 1:** Reservoir heterogeneity coefficient statistics of Badaowan Formation in Seventh Block.

Block	Stratum		Heterogeneity Coefficient of Permeability						
			Qidong 1 Block—East	Qidong 1 Block—West	Qidong 2 Block	Qizhong Block—East	Qizhong Block—West	Qixi Block	Whole Block
Seventh Block	Badaowan Formation	Thin layer	Heterogeneity coefficient of permeability						
		J_1_*b*_5_^1-1^	5.48	4.03	3.34	3.4	5.56	6.86	4.76
		J_1_*b*_5_^1-2^	3.27	3.14	4.01	3.22	3.59	3.61	3.45
		J_1_*b*_5_^1-3^	3.46	3.07	3.96	2.89	3.61	4.51	3.58
		Thin layer	Level difference						
		J_1_*b*_5_^1-1^	30.3	21.7	15.3	13.7	27.5	12	19.6
		J_1_*b*_5_^1-2^	12.4	9.9	7.4	9.5	11.3	9.1	10.1
		J_1_*b*_5_^1-3^	13.1	10.3	8.6	9.4	12.9	8.9	10.4
		Thin layer	Coefficient of variation						
		J_1_*b*_5_^1-1^	0.62	0.61	0.55	0.6	0.63	0.68	0.61
		J_1_*b*_5_^1-2^	0.54	0.52	0.57	0.53	0.56	0.55	0.54
		J_1_*b*_5_^1-3^	0.64	0.65	0.62	0.67	0.61	0.66	0.65

**Table 2:** Parameters of multilayer heterogeneous model.

Sample Information	Porosity/%	Permeability/mD
Low-permeability layer	29.6	37.9
Central permeability layer	31.3	102
High-permeability layer	30.7	538

**Table 3:** Properties and composition of modelled formation water.

Type	Total Mineralisation (mg/L)	Ionic Concentration (mg/L)					
		Cl^−^	HCO_3_^−^	SO_2_^−^_4_	Na^+^	Mg^2+^	Ca^2+^
CaCl_2_	12,714	4961.71	2886.03	84.04	4435.75	21.24	37.09

**Table 4:** Rock sample parameters and experimental grouping.

Level Difference	Combination	Core Number	Porosity (%)	Permeability (mD)	Permeability Contrast	Polymer Formulation	Note
±2	1	02-10A	18.6	79.9	1.75	500 × 10^4^ 0.10% HPAM + 0.2% KPS	
		02-51A	20.9	140			
	2	04-7D	22.7	80.2	1.77	800 × 10^4^ 0.10% HPAM + 0.2% KPS	
		02-6A	23.1	142			
	3	04-4C	21.5	84.1	1.76	1200 × 10^4^ 0.10% HPAM + 0.2% KPS	
		04-1D	23.3	148			
	4	02-4B	24.9	85.1	1.74	1500 × 10^4^ 0.12% HPAM + 0.2% KPS	
		04-5F	21.8	148			
±3	5	02-4D	23.9	85.7	2.65	500 × 10^4^ 0.10% HPAM + 0.2% KPS	
		01-26A	21.6	227			
	6	02-7C	21.3	91.8	3.43	800 × 10^4^ 0.10% HPAM + 0.2% KPS	
		02-45B	19.4	315			
	7	04-4B	20.3	93.1	4.02	1200 × 10^4^ 0.10% HPAM + 0.2% KPS	
		02-46A	23.6	374			
	8	04-1E	20.4	119	3.24	1500 × 10^4^ 0.12% HPAM + 0.2% KPS	
		01-22C	24.1	386			
±6	9	02-4C	20.9	95.1	6.26	500 × 10^4^ 0.10% HPAM + 0.2% KPS	
		01-27D	22.0	595			
	10	02-10C	22.6	106	6.01	800 × 10^4^ 0.10% HPAM + 0.2% KPS	
		02-46D	19.5	637			
	11	A3	21.32	88.3	6.09	1200 × 10^4^ 0.10% HPAM + 0.2% KPS	Plus MRI and NMR
		01-21C	25.41	538.4			
	12	04-10D	23.552	119	5.42	1500 × 10^4^ 0.12% HPAM + 0.2% KPS	Plus MRI and NMR
		01-29	24.639	645			

**Table 5:** Rock sample parameters and experimental grouping.

Level Difference	Core	Porosity (%)	Permeability (mD)	Pore Volume (cm^3^)	Bound Water Saturation (%)	Water Flooding Efficiency (%)	Recovery After SP Flooding (%)	SP Flooding Increase Rate (%)	Parallel Water Flooding Efficiency (%)	Recovery After Parallel SP Flooding (%)	Parallel SP Flooding Improvement (%)	Binary System Formulations
±2	02-10A	18.6	79.9	5.729	40	47.67	56.1	8.43	48.24	57.86	9.62	500 × 10^4^ 0.10% + 0.2% KPS
	02-51A	20.9	140	6.558	35.2	48.71	59.3	10.59				
	04-7D	22.7	80.2	5.917	39.7	39.8	56.62	16.82	45.2	59.72	14.52	800 × 10^4^ 0.10% + 0.2% KPS
	02-6A	23.1	142	5.769	37.6	50.56	62.81	12.25				
	04-4C	21.5	84.1	6.853	38.2	42.27	56.2	13.93	42.66	55.95	13.29	1200 × 10^4^ 0.10% + 0.2% KPS
	04-1D	23.3	148	7.918	34.2	42.99	55.66	12.67				
	02-4B	24.9	85.1	5.52	38.2	41.17	48.53	7.36	42.3	52.36	10.06	1500 × 10^4^ 0.12% + 0.2% KPS
	04-5F	21.8	148	5.887	34.5	43.31	55.76	12.45				
±3	02-4D	23.9	85.7	6.469	39.6	38.39	54	15.61	41.97	53.76	11.79	500 × 10^4^ 0.10% + 0.2% KPS
	01-26A	21.6	227	5.621	42.2	45.66	53.53	7.87				
	02-7C	21.3	91.8	5.779	37.4	37.04	53.9	16.86	43.85	56.81	12.96	800 × 10^4^ 0.10% + 0.2% KPS
	02-45B	19.4	315	6.419	33.8	49.65	59.3	9.65				
	04-4B	20.3	93.1	6.579	38.9	36.32	57.96	21.64	43.28	61.36	18.08	1200 × 10^4^ 0.10% + 0.2% KPS
	02-46A	23.6	374	5.759	31.8	50.41	64.85	14.44				
	04-1E	20.4	119	5.986	38.2	32.17	41.63	9.46	39.21	54.38	15.17	1500 × 10^4^ 0.12% + 0.2% KPS
	01-22C	24.1	386	5.266	33.7	46.69	67.91	21.22				
±6	02-4C	20.9	95.1	7.997	39.1	20.84	27.41	6.57	33.25	43.38	10.13	500 × 10^4^ 0.10% + 0.2% KPS
	01-27D	22	595	7.849	31.8	46.7	57.91	11.21				
	02-10C	22.6	106	5.571	37.1	25.68	34.25	8.57	42.76	51.52	8.76	800 × 10^4^ 0.10% + 0.2% KPS
	02-46D	19.5	637	5.436	37	55.34	66.12	10.78				
	A3	21.32	88.26	5.649	37	26.87	35.23	8.36	37.47	50.47	13	1200 × 10^4^ 0.10% + 0.2% KPS
	01-21C	25.41	538.39	6.325	21.6	45.25	61.37	16.12				
	04-10D	23.552	119	6.077	40	22.67	33.9	11.23	35.59	46.82	11.23	1500 × 10^4^ 0.12% + 0.2% KPS
	01-29	24.639	645	6.563	26.7	45.32	56.48	11.16				

**Table 6:** Core flooding oil recovery versus water content curve.

Gradation	±2	±3	±6
500 × 10^4^ 0.10% + 0.2%KPS	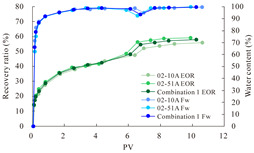	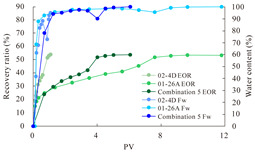	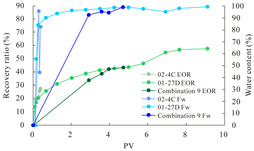
800 × 10^4^ 0.10% + 0.2%KPS	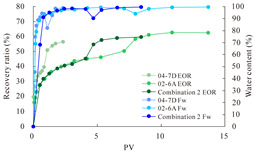	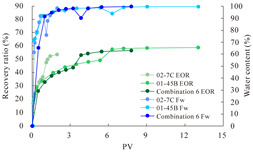	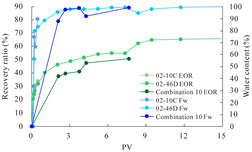
1200 × 10^4^ 0.10% + 0.2%KPS	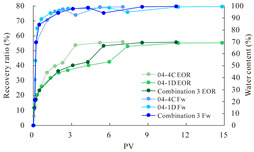	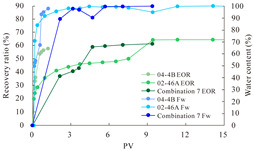	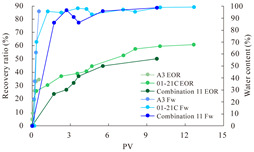
1500 × 10^4^ 0.12% + 0.2%KPS	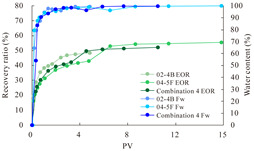	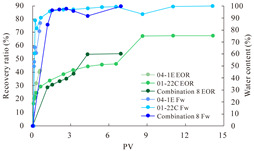	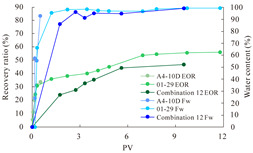

**Table 7:** Comparison of magnitude of recovery enhancement by SP binary composite flooding.

Gradation	±2	±3	±6
500 × 10^4^ 0.10% + 0.2%KPS	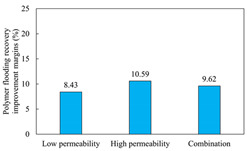	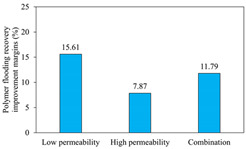	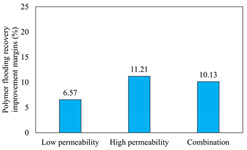
800 × 10^4^ 0.10% + 0.2%KPS	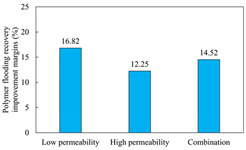	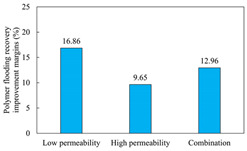	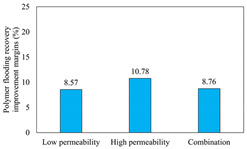
1200 × 10^4^ 0.10% + 0.2%KPS	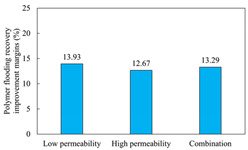	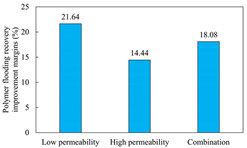	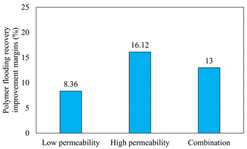
1500 × 10^4^ 0.12% + 0.2%KPS	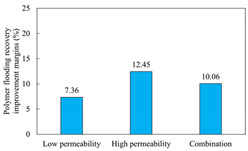	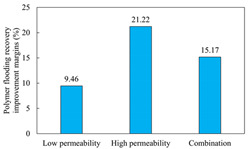	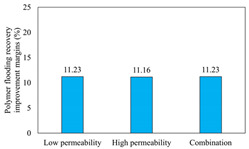

**Table 8:** Microscopic pore movement patterns of rock cores.

Combination	Core	*T*_2_ Distribution Curve	Pore Radius (μm)	Pore Throat Distribution (%)	Oil Content by Volume (%)			Degree of Crude Oil Utilisation (%)		Contributions to Water Flooding Recovery (%)	Contribution of SP Flooding to Recovery Enhancement (%)
					Saturated Oil	Water Flooding	SP Flooding	Water Flooding	SP Flooding		
Combination 11	A3 (low permeability)	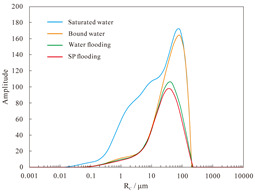	<0.1	5.95	1.32	1.27	1.15	3.50	12.64	0.24	4.31
			0.1~1	27.22	5.64	5.18	5.11	8.19	9.29	2.38	2.21
			1~5	28.28	18.86	17.16	16.72	9.01	11.33	8.75	15.63
			5~20	33.92	32.60	18.33	16.50	43.77	49.38	73.48	65.52
			20~50	4.64	4.63	1.69	1.34	63.58	71.01	15.15	12.33
			>50	0.00	0.00	0.00	0.00	0.00	0.00	0.00	0.00
	01-21C (high permeability)	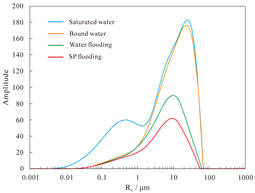	<0.1	5.85	0.70	0.68	0.65	3.07	7.76	0.06	0.27
			0.1~1	19.08	5.34	5.10	4.44	4.40	16.80	0.67	5.34
			1~5	17.81	15.04	12.05	8.47	19.88	43.68	8.60	28.89
			5~20	32.15	30.92	18.09	12.34	41.49	60.09	36.88	46.45
			20~50	22.80	22.61	5.98	3.76	73.57	83.35	47.83	17.87
			>50	2.31	2.25	0.18	0.03	92.05	98.57	5.96	1.19
Combination 12	04-10D (low permeability)	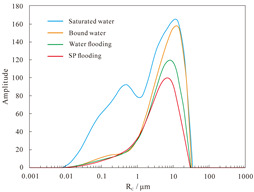	<0.1	9.53	1.52	1.35	1.45	11.74	35.8	0.88	−1.48
			0.1~1	27.33	5.74	5.38	5.54	6.28	3.46	1.77	−2.37
			1~5	25.38	17.58	15.79	15.03	10.17	14.5	8.78	11.27
			5~20	31.95	29.93	21.78	16.49	27.21	43.34	40.03	78.48
			20~50	5.82	5.24	2.11	1.16	59.77	77.88	15.39	14.09
			>50	0	0	0	0	0	0	0	0
	01-29 (high permeability)	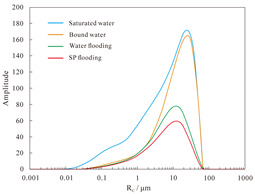	<0.1	3.52	0.69	0.47	0.44	31.52	35.80	0.66	0.36
			0.1~1	14.09	4.62	3.92	3.52	15.11	23.88	2.10	4.96
			1~5	22.72	11.59	10.46	8.61	9.74	25.65	3.40	22.56
			5~20	33.40	29.71	17.93	13.78	39.65	53.63	35.49	50.81
			20~50	24.34	24.44	6.90	5.28	71.77	78.40	52.84	19.83
			>50	2.23	2.19	0.36	0.24	83.36	88.86	5.51	1.48

**Table 9:** Flooding efficiency of multilayer heterogeneity modelling.

Model	Waterflooding Recovery Factor/%	Recovery After Binary Injection/%	Recovery Factor After Secondary Water Flooding/%
Low-permeability layer	9.02	25.90	29.05
Medium-permeability layer	29.35	52.36	56.23
High-permeability layer	51.98	60.46	61.35
Overall model	40.10	51.20	53.68

**Table 10:** Layered flooding efficiency.

Serial Number	Layer Number	Oil Saturation/%	Water Flooding Oil Recovery/%	Improvement in SP Binary System/%
1	First layer	74.3	55.8	63.1
2	Second layer	75.7	60.4	65.6
3	Third layer	62.2	39.0	52.1

**Table 11:** Microscopic pore mobilisation patterns of first-layer cores.

Layer Number	*T*_2_ Distribution Curve	Pore Radius (μm)	Pore Throat Distribution (%)	Oil Content by Volume (%)			Degree of Crude Oil Utilisation (%)		Contributions to Water Flooding Recovery (%)	Contribution of SP Flooding to Recovery Enhancement (%)
				Saturated Oil	Water Flooding	SP Flooding	Water Flooding	SP Flooding		
First layer	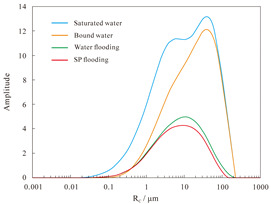	<0.1	0.56	0.07	0.07	0.07	7.14	4.41	0.01	−0.05
		0.1~1	9.71	2.87	2.66	2.62	7.39	8.78	0.49	1.05
		1~5	24.56	13.66	8.95	8.49	34.53	37.88	10.95	11.98
		5~20	26.74	21.55	11.29	9.77	47.61	54.66	23.81	39.82
		20~50	19.75	18.15	5.77	4.86	68.18	73.24	28.73	24.02
		>50	18.69	18.03	2.53	1.64	86.00	90.90	36.00	23.19
Second layer	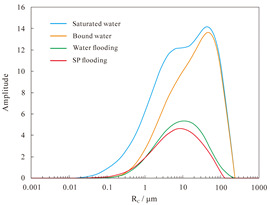	<0.1	0.83	0.03	0.03	0.03	8.45	5.40	0.01	−0.02
		0.1~1	9.96	2.81	2.01	2.43	28.39	13.51	1.74	−10.52
		1~5	22.97	13.16	8.44	7.80	35.85	40.75	10.31	16.23
		5~20	26.02	21.14	11.05	9.49	47.74	55.10	22.05	39.13
		20~50	19.15	18.03	5.80	4.57	67.84	74.64	26.73	30.80
		>50	21.08	20.59	2.67	1.70	87.05	91.76	39.17	24.38
Third layer	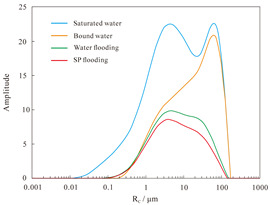	<0.1	1.98	0.02	0.02	0.02	20.22	15.73	0.02	−0.02
		0.1~1	13.81	3.75	3.49	3.26	6.86	12.89	0.95	3.89
		1~5	27.28	12.94	11.55	10.33	10.73	20.19	5.11	21.07
		5~20	24.32	16.08	11.20	9.28	30.34	42.25	17.96	32.95
		20~50	15.18	13.78	6.31	4.73	54.19	65.68	27.50	27.27
		>50	17.42	16.17	3.00	2.14	81.42	86.75	48.46	14.85

## Data Availability

The data are not publicly available due to the need for further relevant research, but they are available on request from the corresponding author.
